# Rapidly Progressive Glomerulonephritis Secondary to Immunoglobulin M-associated Monoclonal Gammopathy of Renal Significance: A Report of a Rare Case

**DOI:** 10.7759/cureus.61937

**Published:** 2024-06-08

**Authors:** Andres E Prieto-Torres, Daniel H Rodriguez-Peralta

**Affiliations:** 1 Internal Medicine, Hospital Militar Central, Bogotá, COL; 2 Nephrology, Hospital Militar Central, Bogotá, COL

**Keywords:** immunoglobulin m, rapidly progressive glomerulonephritis, monoclonal gammopathy of renal significance (mgrs), glomerulonephritis (gn), paraproteinemia

## Abstract

Monoclonal gammopathy of undetermined significance (MGUS) is a premalignant condition characterized by monoclonal paraprotein production, with IgM and non-IgM variants. While IgM MGUS is often associated with lymphoid neoplasms, non-IgM MGUS can progress to multiple myeloma. Comorbidities include bone mineral density loss and renal complications, such as monoclonal gammopathy of renal significance (MGRS) and peripheral neuropathy. Cardiovascular risks are also elevated. Despite its significance, MGUS often goes undiagnosed due to its asymptomatic nature and overlap with age-related comorbidities. We present a case of IgM MGRS manifesting as rapidly progressive glomerulonephritis, highlighting the diagnostic challenges and clinical implications of MGUS-associated complications.

## Introduction

Monoclonal gammopathy of undetermined significance (MGUS) is a premalignant condition characterized by the presence of a monoclonal paraprotein derived from immunoglobulin (Ig). It can be classified into IgM MGUS and non-IgM MGUS, depending on the responsible cellular clone [1]. In most cases, IgM MGUS can develop in the context of lymphoid neoplasms, especially Waldenström macroglobulinemia as well as with non-Hodgkin lymphomas and chronic lymphocytic leukemia. Non-IgM MGUS is derived from mature plasma cells and can progress to multiple myeloma; in a minority of patients, MGUS can be identified as having only light chains, meaning that the isolated secretion is of kappa and lambda light chains of Igs. Depending on the nature of the infiltration of these light chains, there may be an infiltration of light chain amyloidosis or light chain deposition disease [2].

MGUS typically occurs in approximately 3% of individuals over 50 years of age, with an average onset age of around 72 years, more commonly in men and specifically in the Afro-Caribbean population. The annual risk of progression to malignant conditions is approximately 1% [3]. Specifically, IgM MGUS is defined by serum concentrations less than or equal to 3 g/dL, fewer than 10% plasma cells in the bone marrow, and the absence of constitutional symptoms or symptoms or signs of hyperviscosity, anemia, or lymphadenopathy [1]. There are multiple comorbidities commonly associated with MGUS. Population studies show an association between MGUS and low bone mineral density as well as high cardiovascular risk, and there is an increased risk of arterial involvement, venous thromboembolism, and structural cardiac disorders, requiring strict monitoring [4]. Peripheral neuropathy describes the impairment of somatic, enteric, or autonomic neurons outside the central nervous system. The association with MGUS has been identified in approximately 5% of patients [5]. 

Monoclonal gammopathy of renal significance (MGRS) was described in 2012 and encompasses all clonal B-cell precursor or plasma cell disorders that secrete a nephrotoxic monoclonal paraprotein. Monoclonal gammopathy is associated with low levels of monoclonal paraprotein, which do not form full chains but have the capacity to create toxic amyloid multimers and amyloid fibrils from Ig light chain fragments or even, during the degradation process, form crystals as cytoplasmic inclusions in the tubular epithelium. It is now known that monoclonal Igs can dysregulate the alternative complement pathway through autoantibody-like activity against factor H. Given this, renal involvement can be multifaceted; however, the most common manifestations are cast nephropathy and amyloid deposition in the glomerulus. Less frequently and more rarely, there are proliferative and extracapillary predominant forms [6].

Within Ig deposit diseases, there are organized Ig deposits such as fibrillar (light chain Igs (AL), heavy chain Igs (AH), heavy and light chain Igs (ALH), and monoclonal fibrillar glomerulonephritis), microtubular (cryoglobulinemia types I and II and immunotactoid glomerulopathy), and crystalline deposit inclusion (crystalline light chain tubulopathy (LCTP), histiocytosis by crystal deposition, and cryoglobulinemic glomerulonephritis). In addition, there are disorders with unorganized deposits, including monoclonal immunoglobulin deposition diseases (MIDD) and proliferative glomerulonephritis with immunoglobulin deposits (PGNMID), as well as tubular disorders like Fanconi syndrome. The most serious complication of MGRS is end-stage renal disease, occurring in up to 22% of patients, with an approximate median of 30.3 months [6].

Up to 90% of MGUS cases go undiagnosed, despite being considered a premalignant condition of multiple myeloma and other lymphoproliferative disorders. Often, distinguishing MGUS as the cause of the various described organic involvement patterns is difficult, especially given the age group at presentation and the comorbidities of these patients. Here, we present a case of IgM MGRS presenting as rapidly progressive glomerulonephritis (RPGN).

This article was previously presented as a meeting abstract and e-poster at the 2024 World Congress of Nephrology on April 13-16, in Buenos Aires, Argentina.

## Case presentation

A 69-year-old female patient with a medical history of hypertension, diabetes mellitus, bipolar affective disorder, and stage 3B chronic kidney disease frequently sought emergency care due to a clinical picture characterized by dyspnea and progressive edema in the lower limbs. During her initial visits to the service, a brain natriuretic peptide test was performed, which was found to be elevated, along with an echocardiogram that showed diastolic dysfunction, leading to a diagnosis of heart failure with preserved ejection fraction, managed by the internal medicine department with diuretics and foundational therapy properly chosen for her.

However, during hospital admissions from April to July, there was evidence of progressive deterioration in renal function, the development of nephrotic-range proteinuria, and anemia, all associated with difficulty in controlling blood pressure (Table 1). Investigations were conducted for secondary hypertension, including renal artery Doppler, aldosterone levels, and plasma renin activity, all of which returned normal results. Acute kidney injury was progressing, suggesting a rapidly progressive course. Therefore, a biopsy was performed to confirm the diagnosis of RPGN based on histological findings.

**Table 1:** Laboratory parameters from the present case ND: no data; RBC: red blood cells

Test (unit)	April 13	July 4	Normal values
Leukocytes (×10^9^/L)	5.46	4.62	4.5-11.3
Neutrophils (×10^9^/L)	2.89	2.05	2.2-8.4
Lymphocytes (×10^9^/L)	1.83	1.69	0.9-4.5
Platelets (×10^9^/L)	143	82	150-450
Hemoglobin (g/dL)	7.9	8.8	12.1-16.6
Hematocrit (%)	24	25	35-49
Total serum proteins (g/dL)	6.59	ND	6.6-8.7
Creatinine (mg/dL)	1.47	4.02	0.6-1.1
Urea nitrogen (mg/dL)	29	69	8-23
Urinalysis	Color: yellow. Density: 1.008. pH: 6.0. Proteins: 150 mg/dL. Hem: 10 Ery/uL. Sediment: RBC 0 XC	Color: Yellow. Density: 1.019. pH: 5.0. Proteins: 500 mg/dL. Hem: 25 Ery/uL. Sediment: RBC 8-12 XC	ND
24-hour urine protein test	1321 mg/24H	5242 mg/24H	ND

Tests for infectious etiologies, including hepatitis C (HCV), hepatitis B (HBV), human immunodeficiency virus (HIV), and syphilis, came back negative. Regarding immune etiologies, studies with anti-neutrophil cytoplasmic antibodies (ANCA) and anti-DNA were also negative. Anti-nuclear antibodies (ANA) were positive with titers of 1/80 and an AC-28 pattern. Protein electrophoresis showed a polyclonal gamma peak. However, immunofixation revealed a monoclonal peak of IgM heavy chains and lambda light chains with normal serum Ig levels; urine immunofixation showed the elimination of both kappa and lambda chains, both free and bound, all with the appearance of a paraprotein.

**Figure 1:**
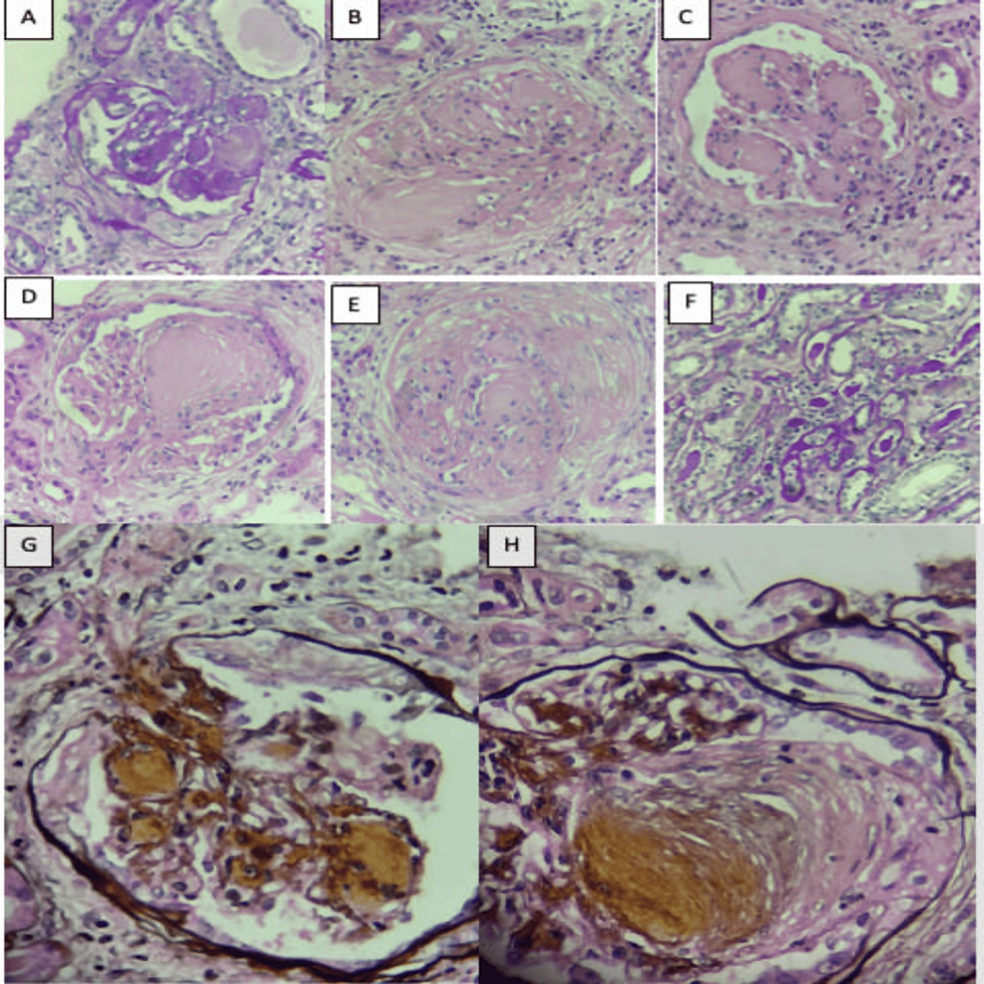
Renal biopsy pathology A-H: Hematoxylin and eosin staining 40×-PAS staining 40×: Glomeruli exhibit diffuse enlargement without hypercellularity, weakly positive with PAS, and negative with silver methenamine; associated foamy histiocytes suggesting the presence of deposits. Mesangium shows other areas with nodular appearance that are positive with PAS and silver, consistent with diabetic nephropathy. Focal extracapillary hypercellularity (fibrocellular crescents). Tubular atrophy of 60%, no evidence of tubulitis, preserved tubular epithelium, hyaline casts, interstitial fibrosis of 50%, and mononuclear interstitial inflammation of 20%. Direct immunofluorescence reported deposits of IgM and kappa light chains PAS: periodic acid-Schiff

In light of these findings, a bone marrow biopsy was conducted, which did not reveal clonal plasma cells, effectively ruling out multiple myeloma, Waldenström macroglobulinemia, and marginal zone lymphoma as potential causes. As a result, it was concluded that the patient likely had a MGRS. With this presumptive diagnosis, a renal biopsy was performed, where the presence of widening without mesangial hypercellularity or mesangiolysis is observed, along with areas of nodular appearance that are negative with methenamine silver stain and weakly positive with periodic acid-Schiff (PAS) stain, with associated histiocytes raising the suspicion of deposits. Additionally, other areas with nodular appearance are noted, which are positive with PAS and silver stains, favoring associated sclerosis. Furthermore, extracapillary hypercellularity (crescents) is observed in three glomeruli, of which two were fibrocellular and one was fibrous. Immunofluorescence revealed the presence of IgM in the mesangium and glomerular basement membrane, as well as C1q, with kappa clonality at the same location, with some cylinders highlighting this immunofluorescence. Unfortunately, electron microscopy processing was not possible due to tissue exhaustion (Figure 1).

Pulse therapy with 500 mg of methylprednisolone over a 72-hour period was administered, but unfortunately, it did not yield any improvement. The hematology department considered initiating a VCD (bortezomib, dexamethasone, cyclophosphamide) regimen. Subsequently, the patient's renal function began to show signs of improvement.

## Discussion

Glomerular diseases encompass a broad group of pathologies determined by age, sex, and the race of patients, with different forms of presentation. Among these forms of presentation, RPGN is often the manifestation of severe glomerular damage, defined as a deterioration of renal function measured through serum creatinine levels or a decrease in urinary output associated with active urinary sediment over a period of a few weeks to a few months, with histopathological evidence of crescent formation [7]. In the etiological study approach of this presentation, the goal is to rule out diseases from group I mediated by antibodies against the glomerular basement membrane (Goodpasture syndrome or isolated glomerulonephritis), group II where the etiology is related to the formation of immune complexes with secondary deposits, usually due to infectious pathologies, such as HCV, HBV, and HIV, or systemic pathologies, like systemic lupus erythematosus (SLE), and less commonly IgA nephropathy (accounting for approximately 15-20% of total cases), and, finally, those defined as pauci-immune from group III, where the systemic vasculitis with renal involvement are grouped, accounting for nearly 80% of total causes [8]. 

In our patient, an investigation was focused on the most common causes in line with their clinical presentation, sequentially ruling them out. The only positive result was obtained in ANAs. However, literature reports indicate that finding these levels of antibodies in healthy populations is common, with up to 15% having these antibodies. The proposed cutoff point for entry into autoimmune pathology studies, 1/80, generally offers a sensitivity of 97.8% with a poor specificity of up to 74.7% [9]. For this reason, adjusting the cutoff point to 1/160 has been proposed, although it is generally rejected due to a significant decrease in test sensitivity. To complete the algorithm, anti-DNA measurement was performed with a negative report, ruling out autoimmune pathologies as the cause in the current case. 

The current literature review shows that several case reports have been found regarding renal involvement due to monoclonal gammopathies. In the first of these reports, proliferative glomerulonephritis with IgG deposits and undetected kappa light chains in electrophoresis and immunofixation is described, possibly secondary to their low quantity and rapid glomerular fixation, attributed to an immune response to some self or external antigen [10]. A second case report documented a patient with renal injury, proposing paraproteinemia as a trigger for complement activation or dysregulation and neutrophil activation, leading to a pauci-immune, ANCA-negative mechanism due to the absence of renal deposits [11]. Finally, a third case of proliferative glomerulonephritis with evidence of IgG3 deposits is described, with its nephrotoxic effects attributed to its high molecular weight, positive charge, and wide complement-fixing capacity. What's unique about this case is the long-term follow-up of the patient, demonstrating that the disease course is slow and can generally have a good prognosis without immunosuppressive treatment [12].

In our specific case, the patient's comorbidities do not explain the rapid progression of her renal failure. Hypertension was eventually controlled with adjustments to antihypertensive medical management, and her diabetes mellitus was adequately controlled, which does not explain the development of nephrotic-range proteinuria or the progression of her kidney disease. The only possible etiology identified after the sequential diagnostic process of study and exclusion was the deposition of IgM and kappa light chains at the level of the glomerular basement membrane and mesangium, leading to complement activation through the classical pathway, as evidenced by the additional C1q marker observed in immunofluorescence, indicating a response to common molecular patterns associated with damage [13]. This suggests the presence of an undetected internal or external antigen that triggered the sequence of events in the patient, as suggested in previous case reports.

Finally, it should be emphasized that there is no consensus on when to initiate treatment in patients with MGRS. Each case should be individualized to determine whether to adopt a "wait and see" approach or to proceed with a specific treatment.

## Conclusions

We report the case of a patient with histological evidence consistent with immunoglobulin M-associated MGRS presenting unusually as rapidly progressive renal failure. After a detailed review of the current literature, there have been no reported cases with similar presentations. While there are documented cases of paraproteinemia leading to the development of MGRS, the etiology in those cases was either pauci-immune or associated with IgG deposits, making our case extremely rare.
